# Symbiotic Sensing for Energy-Intensive Tasks in Large-Scale Mobile Sensing Applications

**DOI:** 10.3390/s17122763

**Published:** 2017-11-29

**Authors:** Duc V. Le, Thuong Nguyen, Hans Scholten, Paul J. M. Havinga

**Affiliations:** 1 Pervasive Systems Group, Department of Computer Science, University of Twente, Drienerlolaan 5, 7522 NB Enschede, The Netherlands; hans.scholten@utwente.nl (H.S.); p.j.m.havinga@utwente.nl (P.J.M.H.); 2 The Australian e-Health Research Centre, CSIRO, Herston, Queensland 4029, Australia; thuong.nguyen@csiro.au

**Keywords:** participatory sensing, opportunistic sensing, success probability, energy consumption, mobile sensing, massive sensing

## Abstract

Energy consumption is a critical performance and user experience metric when developing mobile sensing applications, especially with the significantly growing number of sensing applications in recent years. As proposed a decade ago when mobile applications were still not popular and most mobile operating systems were single-tasking, conventional sensing paradigms such as opportunistic sensing and participatory sensing do not explore the relationship among concurrent applications for energy-intensive tasks. In this paper, inspired by social relationships among living creatures in nature, we propose a symbiotic sensing paradigm that can conserve energy, while maintaining equivalent performance to existing paradigms. The key idea is that sensing applications should cooperatively perform common tasks to avoid acquiring the same resources multiple times. By doing so, this sensing paradigm executes sensing tasks with very little extra resource consumption and, consequently, extends battery life. To evaluate and compare the symbiotic sensing paradigm with the existing ones, we develop mathematical models in terms of the completion probability and estimated energy consumption. The quantitative evaluation results using various parameters obtained from real datasets indicate that symbiotic sensing performs better than opportunistic sensing and participatory sensing in large-scale sensing applications, such as road condition monitoring, air pollution monitoring, and city noise monitoring.

## 1. Introduction

Smartphones with integrated sensors have enabled the development of low-cost and reliable large-scale sensing systems including personal sensing [1,2,3,4], social behavior sensing [5,6,7,8], environmental monitoring [9,10,11,12], smart transportation and road monitoring [13,14,15,16], electromagnetic monitoring [17], radiation monitoring [18], and event monitoring [19]. Since smartphones are consumer devices, a sensing service design should consider a user’s role in performing sensing tasks: data collection, analysis, verification, and sharing. The two most popular sensing paradigms are opportunistic sensing [20] and participatory sensing [21]. Opportunistic sensing executes sensing tasks transparently to the users. Conversely, participatory sensing requests the users to interact with the application to perform sensing tasks.

Although these sensing paradigms have been widely used in large-scale sensing systems, they do not focally address the energy consumption on smartphones. One of the reason might be there were not so many applications and sensors on smartphones as there are today. With the significant development of smartphone technologies, more sensors will be integrated including sophisticated and power-hungry sensors such as gas sensors [22]. Consequently, data harvesting through smartphones invokes a variety of challenges related to the limited available battery capacity. Additionally, potential privacy breaches when using smartphone applications are of increasing concern in society [23]. Therefore, it is hard to convince users to take part in sensing tasks, especially when the application is not of their interest. A common solution for this problem is an incentive for participants [24,25,26]. However, it is costly to recruit a large number of participants. Therefore, these energy and privacy constraints are still challenging when deploying large-scale sensing applications on smartphones.

Besides the aforementioned constraints, multi-task mobile operating systems also yield the issue of application concurrency. Sensing applications may not be able to access some sensors such as microphones when they are being used by another application. This issue is especially important as the average number of applications per smartphones in 2015 is 36 [27]. A recent report [28] also reveals that the monthly time a person spends on smartphone applications has risen to 63% in two years, from about 23 h in 2012 to about 37.5 h in 2014. Consequently, the possibility of conflict between sensing applications acquiring the same sensing resources will increase. However, existing sensing paradigms do not consider this concurrency issue.

Therefore, there is a demand for new sensing paradigms that not only are transparent to users but also can cope with energy, concurrency, and privacy constraints. Inspired by symbiotic relationships among nature livings, we propose a symbiotic sensing paradigm to realize such an emerging demand. The intuition is that if sensing applications can adapt themselves to the environment where they are installed and run transparently, the total used energy would be significantly reduced. The environment includes other applications, smartphone contexts, user activities and the physical world. In addition, the applications should collaborate to gain off-the-shelf benefits from each other. For example, if two applications can share common resources or derived information, they do not have to waste energy performing the same sensing tasks multiple times. To overcome the concurrency problem and to prevent potential privacy breaches, we develop a cross-sensing service, called SENSILO, to allow sensing applications to share and exchange data, as well as results, in an efficient and secure manner. We make this service publicly available to the research community to deploy symbiotic sensing in real large-scale sensing applications.

We note that symbiotic sensing does not conflict with existing sensing paradigms. Indeed, symbiotic sensing enhances opportunistic sensing in terms of the energy consumption and the conflict of acquiring the same resources. Examples include when a sensor is occupied by one application and cannot be accessed by another application. Therefore, we also propose a hybrid sensing paradigm that combines symbiotic, opportunistic, and participatory sensing for applications that prioritize task completion over energy consumption.

To compare the aforementioned sensing paradigms, we develop mathematical models for evaluating them in terms of *success probability* and *energy consumption* in relation to the total number of smartphones participating in the system. Success probability is the probability of accomplishing a sensing task. Our evaluation model takes into account multiple aspects that have not been addressed in existing evaluation models, such as sensor availability, resource conflict, and task overlap. The models are evaluated with available statistical data and experimental datasets to compare the performance of symbiotic sensing with existing paradigms. Our quantitative analysis shows that symbiotic sensing achieves an equivalent probability of success to others when there are sufficiently large quantity of smartphones participating in the system. This condition is satisfiable by the current proliferation of smartphones. Moreover, our proposed sensing paradigm is more efficient than existing ones in terms of energy consumption.

In short, our main contributions in this work are listed as follows.

1. A comprehensive review of existing sensing paradigms and their applications with 64 high-impact mobile sensing systems.
2. The proposal of symbiotic sensing, a paradigm inspired by symbiotic relationships among living creatures in the nature.
3. Mathematical models for evaluating sensing paradigms that take into account multiple issues encountered in real-world sensing situations.
4. Quantitative evaluation results of sensing paradigms using statistical parameters and available experimental datasets obtained by existing literature.
5. The implementation of SENSILO, a cross-sensing service running on the Android platform to allow the deployment of symbiotic sensing in real world.

The rest of this paper is organized as follows. Section 2 reviews related work in large scale urban sensing. Section 3 discusses the symbiotic relationships among living species in the natural world, which motivates our proposed symbiotic sensing paradigm. Section 4 formulates the problem in terms of evaluation criteria and presents the evaluation models. The sensing paradigms are then quantitatively evaluated in Section 5. Section 6 presents our discussion in regards to key features, privacy issues, and SENSILO implementation. Finally, we conclude our paper in Section 7.

## 2. Background on Urban Sensing

Sensing with mobile device integrated sensors is a new approach that empowers citizens to create a large-scale network to improve the living condition of society. Smartphone owners are encouraged to contribute sensing tasks (e.g., data collection, analysis, verification, and sharing) using their devices for societal and environment information extraction and services, which include healthcare, public safety, environment conservation, and transportation.

In the last decade, a number of studies have developed frameworks, platforms and techniques for various applications with mobile phones using the opportunistic sensing paradigm. Table 1 partially lists the papers that can be found with the keywords including *mobile pervasive computing*, *mobile sensing*, *smartphone-based sensing*, *human-centric sensing*, *urban sensing*, *mobile crowd sensing*, and *mobile crowdsensing*. We use these generic keywords to avoid bias selection of sensing paradigms. With these keywords, we search most relevant literatures in various digital libraries including Google Scholar, IEEEexplore, Web of Science, and Scopus. It is interesting to observe that most current large-scale mobile sensing systems are developed based on either opportunistic sensing [20] or participatory sensing [21]. Only a few systems use hybrid sensing, albeit their approaches simply combine the opportunistic and participatory sensing approaches. Although some recent work, such as [29], have already used communication piggybacking to save energy consumed through the transmission of data to central server, they have not considered the collaboration between the applications, resource sharing and information exchange.

**Figure 1:**
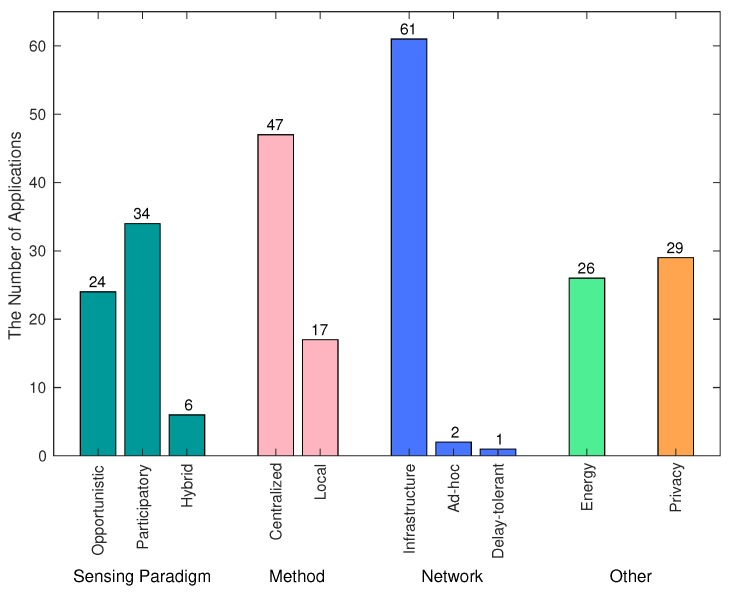
Statistics of the surveyed applications. We classify the applications according to five criteria: sensing paradigm (opportunistic, participatory, and hybrid), analysis method (centralized vs. local), network, energy, and privacy.

Literatures listed in Table 1 are summarized in Figure 1. We classify the applications according to five main criteria: sensing paradigm (opportunistic, participatory, and hybrid), analysis method (centralized vs. local), network, energy, and privacy. The summary numbers show that most current work use either opportunistic or participatory sensing approaches. We hypothesize this is because opportunistic and participatory sensing are plausible in terms of implementation. Moreover, most current work are deployed in a small-scale and controllable experiments. Therefore, they do not seriously encounter the problems of resource conflict and battery consumption, which are confirmed by our evaluations in Section 5.

Figure 1 also shows that 73% of listed studies (47/64) send sampled data to a powerful central server for heavy data processing. Most applications (61/64) send (processed) data directly to a server through infrastructure-based networks such as 3G or WiFi. In addition, only 40% of the studies (26/64) concern energy consumption, and 45% of the studies (29/64) the privacy problem of their systems.

### 2.1. Opportunistic Sensing

**Figure 2:**
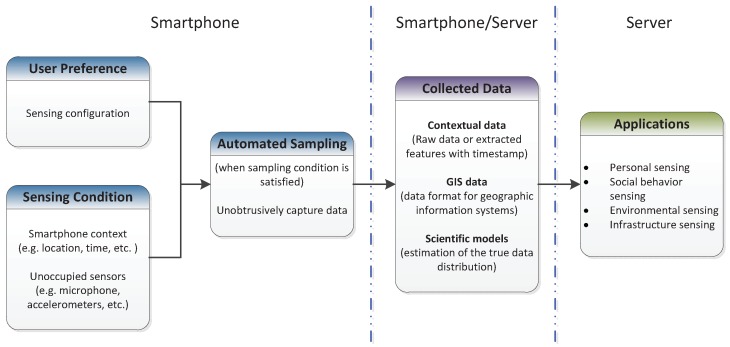
Common architecture for opportunistic sensing.

In opportunistic sensing, the smartphones start performing sensing tasks themselves when the context is *appropriate*, satisfying user preferences, location, time, and sensor availability. Opportunistic sensing systems run unobtrusively such that the users may not be aware of the sensing tasks performed on their devices. Figure 2 illustrates a common architecture for the opportunistic sensing systems. When sensing context is satisfied, sensory data will be unobtrusively captured and analyzed by the application. The collected data will then be sent to a server for further analysis.

Although opportunistic sensing is transparent to users, as data sampling is fully automated, there are still several reasons that may deter users from installing the sensing applications. Opportunistic sensing drains batteries and may disclose personally sensitive information (e.g., a user’s location or context) indirectly when providing sensory data without the permission or awareness of the user. Nevertheless, many users would still accept installing the sensing application in order to participate in opportunistic sensing because they can receive incentives [25,26], or are altruistic by themselves for the greater societal benefit.

However, maintaining transparency is a challenging task since it is technically difficult to determine when the context of the device is suitable for data sampling. An example is to know when the smartphone is out of pocket or bag to collect sound samples for a city noise map application. A common solution is tracking users’ activities and their devices’ context continuously; albeit it is not an easy task.

### 2.2. Participatory Sensing

**Figure 3:**
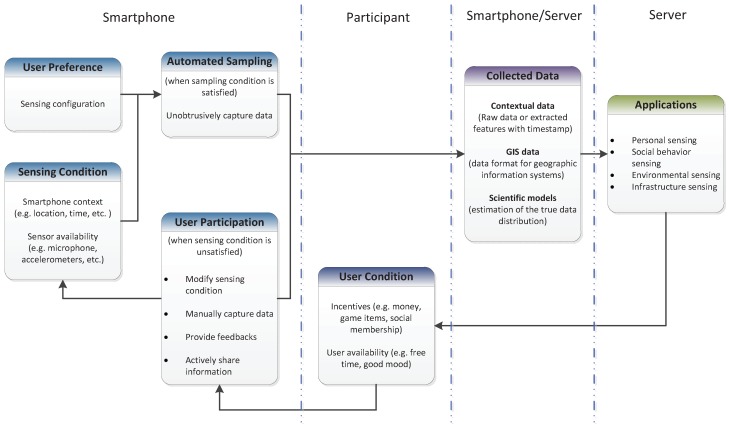
Common architecture for participatory sensing.

In participatory sensing, the user is involved in the sensing process to actively collect and share data. In contrast to opportunistic sensing, participatory sensing places the burden on the user to enhance data collection. Figure 3 illustrates the common architecture of the participatory sensing paradigm. When the sensing condition is *unsatisfied*, the user is requested to cooperate with the smartphone to perform sensing tasks. For example, the user is asked to capture some photos of an event happening where he/she is standing or to validate and share context information retrieved by the application. The higher level of data processing can be done locally on the device or centrally on a powerful server [60].

Since participatory sensing demands the involvement of the users, more users will hesitate to participate and may not allow the application to be installed on their devices, when compared with opportunistic sensing. To this end, participatory sensing should focus on tools and mechanisms that reduce user effort to as little as possible to provide data. Another solution is the provision of some credits, such as social benefits, monetary incentives [73,74], or game items, to the participants to encourage their participation. However, it would be costly to recruit a large number of participants in a large scale sensing system.

## 3. Symbiotic Sensing: Motivation and Definition

In biology, symbiosis refers to relationships between organisms of different species that show an intimate association with each other. A symbiotic relationship requires at least one of the participating species to have a nutritional advantage, usually called the host. In general, symbiosis is categorized into the three following types, depending on the nature of the relationship.

- **Parasitism** is the relationship in which the parasite derives nourishment from the host. It is detrimental to the host. Examples include ticks, fleas, and leeches living on other big animals.
- **Commensalism** is the relationship that benefits only one of the partners. Neither of species is dependent on the other for its existence. Examples include the relationship between porcelain anemone crabs and anemones. The crabs benefit by gaining protection from the anemones.
- **Mutualism** is the relationship in which both partners benefit from each other but they may still be able to live independently. For example, the clown fish gain protection from the anemones. In return, they drop craps of food for the anemones to eat.

In the context of symbiotic relationships, opportunistic sensing and participatory sensing can be seen as parasitism. In opportunistic sensing, sensing applications drain sensing resources from the smartphones, which may be in use by another application. Consequently, smartphone battery is depleted more quickly and other applications may not be able to access the resources. In participatory sensing, sensing applications send requests to the user to obtain data. Users are supposed to manually return their observation or to activate required sensors to collect data. Either way, participatory sensing demands effort from the users. Moreover, in opportunistic and participatory sensing, there might be a conflict when the same sensor is requested by more than one application. Thus, if the sensing application has a lower priority, which is a common situation, the sensing task may not be accomplished.

However, a sensing application can reuse the information or data that has already been obtained by another application. For example, an application aiming to detect a user’s mood can reuse the voice signal sensed by the microphone during phone calls. By doing so, the application consumes very little extra energy. It also does not require the users to perform additional tasks for sensing. More importantly, the sensing application can perform its sensing tasks without any conflict with the phone call application. Note that the exchange can be performed not only between the applications within the same smartphone but also among multiple smartphones in a cooperative manner. If a smartphone does not have a certain type of sensor required for a sensing task, the application can also acquire the data from nearby smartphones that have such sensors by means of commensalism and mutualism. Therefore, if a sensing application is developed using the symbiotic mechanism, it can benefit from the smartphones, other applications, and users without interrupting or draining much extra energy. These advantages would help symbiotic sensing to attract more altruistic people to use the sensing application. To improve the deployment success rate, incentive mechanisms should be explored and implemented [24,25,26,73,74].

Inspired by the aforementioned symbiotic relationships, we propose the symbiotic sensing paradigm that allows sensing applications to reuse resources and information available from other applications to reduce energy consumption. The paradigm also avoids the conflict of requesting the same sensing resources concurrently.

**Definition** **1.** Symbiotic sensing is a sensing paradigm that allows sensing applications to share either resources or sensing results with each other to avoid acquiring or processing the sensory data multiple times. Applications designed with symbiotic sensing do not detriment each other, but they benefit from the association by gaining shared resources and results.

**Figure 4:**
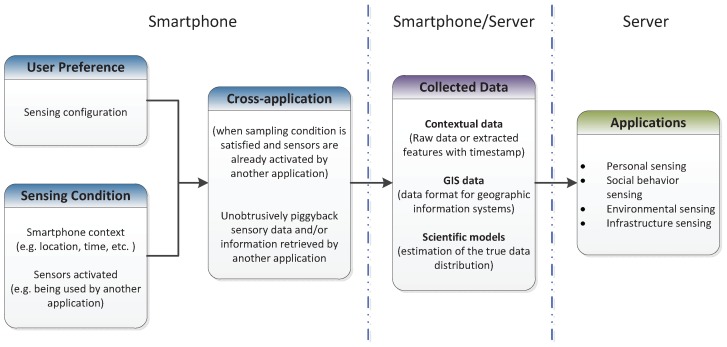
Common architecture for symbiotic sensing.

Figure 4 illustrates a common architecture of the symbiotic sensing paradigm. The key feature is a cross-application layer that allows applications to share sensed information such as contextual data, derived information and models. Unlike the opportunistic sensing paradigm, which performs sampling data only when the sensor is unoccupied, the symbiotic sensing acquires the sensor if and only if it is being used by another application to save energy. This piggybacking behavior of symbiotic sensing is also applied for communication and derived information. For example, an activity recognition application can benefit from a tracking application to enhance its reliability. It is unlikely that a smartphone user is walking when the tracking application identifies that they are moving at a high speed similar to a car.

## 4. Problem Formulation and Evaluation Models

A daunting task for large-scale sensing applications using smartphones is the collection of reliable data in a region of interest by using off-the-shelf smartphone sensors and then sending them to a central server. Some examples of the collected data are environmental noise, temperature, dust particles, carbon dioxide levels, radiation levels, road conditions, and events. For high reliability, data needs to be sampled by at least a number smartphones. However, smartphones are controlled by different users, and functioning in multiple roles, for example, in an application using the smartphones of car drivers to detect road bumps. However not all cars runs via the same roads. Most drivers try to avoid the bumps also. Moreover, some smartphones may run out of battery or the initial sensors may be occupied by mobile games. Therefore, it is a tough challenge to ensure the completion probability of data collection under these various sensing contexts.

Without loss of generality, we formulate the evaluation models for the data collection problem. We assume that data is required to be collected from *C* types of sensors. We further assume that the sensing application is installed on *N* smartphones. The collected data will then be sent to a central server in raw or processed format. To obtain a certain accuracy, each type of sensory data needs to be collected by at least *M* smartphones, where M⩽N.

To be able to choose a suitable sensing paradigm to deploy such sensing application, we need quantitative evaluations to compare the performance of sensing paradigms. Two most important aspects to evaluate a sensing paradigm is the probability of success and energy consumption. Therefore, in this paper, we propose evaluation models on these aspects. Formally, we define success probability and estimated energy consumption in the following subsections.

### 4.1. Probability of Success

A sensing task is considered successful if and only if it is performed by at least *M* smartphones, where *M* is predefined. In reality, the number of required smartphones is typically much smaller that the total number of available smartphones *N*.

**Definition** **2.** Probability of Success of a sensing paradigm is the probability that a sensing task is performed by at least M smartphones to obtain a certain level of accuracy, given a system consisting of N smartphones, M⩽N.

As a sensing task can only be executed under a certain condition, such as sensor availability, smartphone context, and time, let *p* be the probability that the sensing application can perform a sensing task on a single smartphone. The probability of success of a generic sensing paradigm is given in the following lemma.

**Lemma** **1.** *Probability of Success. The success probability of a sensing paradigm is computed as:*

(1) $$P=1-\sum_{k=0}^{M-1}C_{N}^{k}p^{k}{\left(1-p\right)}^{N-k},$$

*where $C_{N}^{k}=\frac{N!}{\left(k!(N-k)!\right)}$ is the number of k-combinations of N elements.*

**Proof.**  The probability that the sensing task is performed by an arbitrary set of *k* smartphones is the joint probability that *k* smartphones perform the sensing task and N−k smartphones do not perform the sensing task, i.e., $p^{k}{\left(1-p\right)}^{N-k}$. In fact, there are $C_{N}^{k}$ combinations where the sensing task is carried out by *k* smartphones. Therefore, the probability that the sensing task is performed by *k* smartphones is given by

$$P\left(k\right)=C_{N}^{k}p^{k}{\left(1-p\right)}^{N-k}.$$

Since the sensing task needs to be performed by at least *M* smartphones, the probability of success is computed as

$$P=1-\sum_{k=0}^{M-1}P(k)=1-\sum_{k=0}^{M-1}C_{N}^{k}p^{k}{\left(1-p\right)}^{N-k}.$$

☐

The rationale behind Lemma 1 is that the probability of success heavily depends on whether a smartphone can perform a sensing task requested by the sensing application, which is represented by *p*. Different sensing paradigms impose different conditions for the smartphone that result in a different sensing probability *p*. Since the performance of smartphones are independent of each other, the probability of success is a joint probability.

The probability that the smartphone can perform a sensing task, denoted by *p*, is assumed to be equal for all the smartphones in Lemma 1. This simplification aims at making the model comprehensible. In real-world applications, the probability *p* can be different among smartphones due to the heterogeneity, e.g., different smartphones models and brands, different users, and different tasks. Under these circumstances, *p* in Lemma 1 can be simply computed by averaging the individual probabilities, which is sufficient for most applications. For a more accurate model of the probability of success, a heterogeneous model should be studied like [75,76].

There are several issues that may affect the chance of a sensing task being performed on a smartphone. For example, the user must agree to take part in the sensing task, the phone must be in a suitable context for data sampling (e.g., at a particular location), the phone must have the required sensor, etc. Therefore, to make the derivation more clearly, we first define a number of elementary probabilities as follows.

**Definition** **3.** Probability of Permission ($P_{p}$) is the probability that a user agrees to take part in sampling data when there is a request.

**Definition** **4.** Probability of User ($P_{u}$) is the probability that a user actually takes part in sampling data, for example, the probability that a user takes their smartphone out of their pocket just to take a photo of the sky for an air monitoring application when the application requests.

**Definition** **5.** Probability of Context ($P_{c}$) is the probability that a smartphone has its context matched with the sampling requirements, for example, when a smartphone is out of the pocket for some other purpose but also can thus be used to record environmental noise.

**Definition** **6.** Probability of Sensor ($P_{s}$) is the probability that a smartphone is integrated with the sensor type required by the sensing task.

**Definition** **7.** Probability of Occupation ($P_{o}$) is the probability that the required sensors are being occupied by another application given the matched context, for example, the percentage of time the user using his/her smartphone to take a picture for himself/herself.

In symbiotic sensing, a phone completes a sensing task only if it owns the required sensor, it is in relevant context, and there is another application using the required sensor. In other words, the probability that it completes the sensing task is a joint probability $p=P_{s}P_{c}P_{o}$. Therefore, the *success probability of symbiotic sensing* is

(2) $$P_{symbiotic}=1-\sum_{k=0}^{M-1}C_{N}^{k}{\left(P_{s}P_{c}P_{o}\right)}^{k}{\left(1-P_{s}P_{c}P_{o}\right)}^{N-k}.$$

On the other hand, in opportunistic sensing, a phone completes a sensing task only if the required sensor is not being used by any other application, making the probability $p=P_{s}P_{c}{\bar{P}}_{o}$, where ${\bar{P}}_{o}=1-P_{o}$ is the complement of $P_{o}$. Therefore, the *success probability of opportunistic sensing* is

(3) $$P_{opportunistic}=1-\sum_{k=0}^{M-1}C_{N}^{k}{\left(P_{s}P_{c}{\bar{P}}_{o}\right)}^{k}\times {\left(1-P_{s}P_{c}{\bar{P}}_{o}\right)}^{N-k}.$$

There are two scenarios in which a sensing task is completed in participatory sensing. When the sensing context is matched, the smartphone may execute the sensing task without help from the user with probability $P_{p}P_{s}P_{c}{\bar{P}}_{o}$. Otherwise, if the sensing context is not matched, the application will request the user to help. However, the user might be reluctant to do so with a probability of $P_{u}$. Therefore, the probability that the smartphone can execute the sensing task with the support from the user is the joint probability $P_{p}P_{s}{\bar{P}}_{c}P_{u}$, where ${\bar{P}}_{c}=1-P_{c}$. Thus, we have the probability that the smartphone can execute the sensing task with participatory sensing as $p=P_{p}P_{s}\left(P_{c}{\bar{P}}_{o}+{\bar{P}}_{c}P_{u}\right)$. Replacing this probability *p* into Equation (1), we have the *success probability of participatory sensing* computed as

(4) $$P_{participatory}=1-\sum_{k=0}^{M-1}C_{N}^{k}{\left[P_{p}P_{s}\left(P_{c}{\bar{P}}_{o}+{\bar{P}}_{c}P_{u}\right)\right]}^{k}\times {\left[1-P_{p}P_{s}\left(P_{c}{\bar{P}}_{o}+{\bar{P}}_{c}P_{u}\right)\right]}^{N-k}.$$

The hybrid sensing paradigm is slightly different to participatory sensing. When the sensing context is matched, the smartphone will sample data regardless of the availability of the required sensor with the support of a resource sharing application service. Additionally, a hybrid sensing application does not request the user to support collecting data if the sensing context is matched. Therefore, the probability that the smartphone can execute the sensing task when the context is matched is $P_{s}P_{c}$. If the sensing context is unmatched, the application will request the user to assist in sampling data. Therefore, the probability that the sensing task can be completed with the assistance of the user is $P_{s}{\bar{P}}_{c}P_{p}P_{u}$. Hence, the possibility that the smartphone can perform the sensing task is the joint probability $p=P_{s}\left(P_{c}+{\bar{P}}_{c}P_{p}P_{u}\right)$. Thus we can define the *success probability of hybrid sensing* as

(5) $$P_{hybrid}=1-\sum_{k=0}^{M-1}C_{N}^{k}{\left[P_{s}\left(P_{c}+{\bar{P}}_{c}P_{p}P_{u}\right)\right]}^{k}\times {\left[1-P_{s}\left(P_{c}+{\bar{P}}_{c}P_{p}P_{u}\right)\right]}^{N-k}.$$

### 4.2. Estimated Energy Consumption

To have a sensing task performed by at least *M* smartphones, the application needs to be installed on *N* smartphones. As the task execution is probabilistic, we need to estimate the total energy consumption of the application on such *N* devices.

**Definition** **8.** Estimated Energy Consumption of a sensing paradigm is the estimated quantity of energy consumed by the application installed on N devices of the system during a unit of time, such that a sensing task is performed by at least M smartphones to obtain a certain level of accuracy, M⩽N.

Energy consumption of a sensing system consists of multiple aspects, e.g., the energy to run the phone and sensors in idle mode, the energy to run the sensors for data collection, and data transmission energy. Therefore, we define the elementary energy consumption as follows.

**Definition** **9.** Idle Energy Consumption ($e_{i}$) is the energy that the application consumes during a unit of time when it is idle, without capturing any data from sensors or doing localization.

As the energy consumed during active mode is typically higher than the energy consumed during idle mode, we define the following types of energy as the extra amount of energy consumed during idle mode.

**Definition** **10.** Sensor Energy Consumption ($e_{s}$) is the extra energy that the requested sensor consumes while performing the sensing task during a unit of time.

Since the sensory data might not be meaningful without the associated location information, we consider energy consumption for localization as one of the key elements to estimate energy consumption.

**Definition** **11.** Localization Energy Consumption ($e_{l}$) is the extra energy that a localization system consumes to update the location information of sampled data during a unit of time.

**Definition** **12.** Communication Energy Consumption ($e_{c}$) is the extra energy that a device consumes to transmit sampled data to another device or a server during a unit of time.

Given the above definitions of elementary energy consumption, we propose the estimated energy consumption for a generic sensing paradigms as follows.

**Lemma** **2.** *Expectation of Energy Consumption. Let p be the probability of a sensing task being performed successfully on a single smartphone in a system of N smartphones. The expectation of the total energy E consumed by the system is given by*

(6) $$\bar{E}=\frac{N+M}{2}p\left(e_{s}+e_{l}+e_{c}\right)+Ne_{i}$$

*where M is the required minimum number of smartphones that perform the sensing task.*

**Proof.**  By definition, *p* is the probability that the phone is in active mode for sensing, which consumes sensor energy $e_{s}$, localization energy $e_{l}$, and communication energy $e_{c}$, on top of idle energy $e_{i}$. This also means that $\left(1-p\right)$ is the probability that the sensing application is in idle mode, which consumes only idle energy $e_{i}$. Therefore, the estimated energy consumed by the application on a single smartphone is given by $p\left(e_{s}+e_{l}+e_{c}+e_{i}\right)+\left(1-p\right)e_{i}$.

If the sensing task is performed by *k* smartphones, the estimated energy consumed by those *k* smartphones is $k\left[p\left(e_{s}+e_{l}+e_{c}+e_{i}\right)+\left(1-p\right)e_{i}\right]$. At the same time, there are another $\left(N-k\right)$ application instances that are in idle mode and consume a quantity of energy equal to $\left(N-k\right)e_{i}$. Thus, the energy consumed by the system when there are *k* smartphones performing the sensing task is given by

$$\begin{array}{cc} E(k) & =k\left[p\left(e_{s}+e_{l}+e_{c}+e_{i}\right)+\left(1-p\right)e_{i}\right]+\left(N-k\right)e_{i}\\ & =kp\left(e_{s}+e_{l}+e_{c}\right)+Ne_{i}. \end{array}$$

As the sensing task succeeds only when it is performed by at least *M* smartphones, we only take into account $E\left(k\right)$ with M≤k≤N. The estimated energy consumption can be one of the values in the set $\left\{E\left(k\right),\;k=M,\cdots ,N\right\}$ with a probability of $\frac{1}{N-M+1}$. Therefore, we have the expectation of energy consumption computed by

$$\begin{array}{cc} \bar{E} & =\sum_{k=M}^{N}\frac{1}{N-M+1}E\left(k\right)\\ & =\sum_{k=M}^{N}\frac{1}{N-M+1}\left[kp\left(e_{s}+e_{l}+e_{c}\right)+Ne_{i}\right]\\ & =\frac{N+M}{2}p\left(e_{s}+e_{l}+e_{c}\right)+Ne_{i}. \end{array}$$

☐

For *symbiotic sensing*, as shown in Equation (2), the probability that the sensing application performs the task is $p=P_{s}P_{c}P_{o}$. We can replace this probability *p* in Equation (6). However, unlike other sensing paradigms, a symbiotic sensing application does not consume extra energy to activate the sensor as it reuses the data sampled by another host application. It can also retrieve the location information recently retrieved by another application, such as Google maps or Facebook. Furthermore, it is possible to piggyback on another application to transmit sampled data without consuming extra power by increasing the bandwidth or data rate as being studied in [77]. Therefore, $e_{s}$, $e_{l}$ and $e_{c}$ in Lemma 2 can be omitted for symbiotic sensing in most cases. In other words, we have the expected energy consumption of symbiotic sensing given by

(7) $${\bar{E}}_{symbiotic}=Ne_{i}.$$

As shown in Equation (3) for *opportunistic sensing*, the probability that the sensing application performs the sensing task is $p=P_{s}P_{c}{\bar{P}}_{o}$. Replacing this probability *p* into Lemma 2, we obtain the expected energy consumption of opportunistic sensing as

(8) $${\bar{E}}_{opportunistic}=\frac{N+M}{2}P_{s}P_{c}{\bar{P}}_{o}\left(e_{s}+e_{l}+e_{c}\right)+Ne_{i}.$$

For *participatory sensing* as can be seen in Equation (4), the probability that the sensing application performs the task is $p=P_{p}P_{s}\left(P_{c}{\bar{P}}_{o}+{\bar{P}}_{c}P_{u}\right)$. Replacing this probability *p* into Lemma 2, we obtain the expected energy consumption of participatory sensing as

(9) $$\begin{array}{c} {\bar{E}}_{participatory}=\frac{N+M}{2}P_{p}P_{s}\left(P_{c}{\bar{P}}_{o}+{\bar{P}}_{c}P_{u}\right)\\ \times \left(e_{s}+e_{l}+e_{c}\right)+Ne_{i}. \end{array}$$

For *hybrid sensing* as shown in Equation (5), the probability that the sensing application performs the sensing task is $p=P_{s}\left(P_{c}+{\bar{P}}_{c}P_{p}P_{u}\right)$. However, the probability that the application executes the sampling task by acquiring sensors is only $P_{s}\left[P_{c}\left(1-P_{o}\right)+{\bar{P}}_{c}P_{p}P_{u}\right]$. For the rest of the probability, $P_{s}P_{c}P_{o}$, the application piggybacks on other applications to gain the power consumption benefit. Therefore, the expected energy consumption of hybrid sensing is given by

(10) $$\begin{array}{c} {\bar{E}}_{hybrid}=\frac{N+M}{2}P_{s}\left[P_{c}\left(1-P_{o}\right)+{\bar{P}}_{c}P_{p}P_{u}\right]\\ \times \left(e_{s}+e_{l}+e_{c}\right)+Ne_{i}. \end{array}$$

## 5. Quantitative Evaluation

In this section, we evaluate symbiotic, opportunistic, participatory and hybrid sensing paradigms in terms of the models given in Section 4. Although our evaluation models are applicable to any kind of sensor, we demonstrate them on two case studies of urban sensing applications using microphones or cameras of smartphones. The reason for choosing to demonstrate on these sensors is that they are the most power-hungry sensors of modern smartphones. Looking at the applications listed in Table 1 (Section 2), we observe that road monitoring [13,14,16,60,72] and noise monitoring [45,47,53,58,59] are quite common applications. Among these works, we select NeriCell [13] (road bump detection) and Ear-Phone [47] (city noise map) as representatives since they have been very well recognized by the urban sensing community. NeriCell had more than 1000 citations and Ear-Phone had more than 500 citations. Moreover, NeriCell is a representative of opportunistic sensing while Ear-Phone is the counterpart of participatory sensing. In these case studies, we evaluate the probability of success and estimated energy consumption using realistic statistical parameters obtained from the literature. For each case, we first evaluate the system with the original sensing paradigm and then evaluate it with the symbiotic or hybrid sensing paradigm. As explained in Section 4.1, the probabilities are assumed to be equal on all the smartphones. When building a real system, the probabilities of smartphones should be averaged when applying our probabilistic models. More realistic models that incorporate the heterogeneity of smartphones like [75,76] are planned in future work. The evaluation models were implemented with Matlab. The source codes that were used to produce the results in this paper can be found at [78].

### 5.1. Case Study 1: Road Bump Detection

NeriCell [13] proposes a smartphone-based sensing system based on the opportunistic sensing paradigm that aims to detect potholes, bumps, braking, and honking. Besides accelerometers, GSM radio, and/or GPS sensors, NeriCell uses microphones of smartphones to detect honks and identify noisy and chaotic traffic conditions like that at an unregulated intersection. The number of detected honks, together with corresponding locations and time, are sent to a data server for further processing.

To evaluate the system in this use case, we obtain the elementary probabilities such as the probability of sensor $P_{s}$ or probability of context $P_{c}$ from the literature. As each smartphone has at least one microphone, we set $P_{s}=1$ for the microphone sensor type. Since microphones are power-hungry, the NeriCell system continuously monitors the Global System for Mobile Communications (GSM) radio and accelerometer to trigger the microphones when needed, such as when braking is detected. If significant levels of braking as well as honking are detected, it might indicate traffic chaos. Such a condition can be considered as an element of context matching for sampling data. During their experiments in Bangalore and Seattle, microphones were active 5% of the time. In addition, when performing opportunistic sensing in real-world situations, smartphones also need to be outside pockets for reliable audio measurements [79]. In 2012, Bristons spent an average of 90 min per day on their smartphones [80]. Although a smartphone can be out of pocket even if it is not in use (such as when it is left idle on a table) we use this statistical number as an approximate probability of the context matching. Therefore, the probability that the context of a smartphone is matched with the sensing condition is given by

(11) $$\begin{array}{cc} P_{c} & =P_{HaveBump}\times P_{OutPocket}\\ & =0.05\times (90/(24\times 60))=0.0031. \end{array}$$

Moreover, in [13], microphones on smartphones are assumed to be always ready to perform a sensing task, but this assumption does not hold in real-world scenarios due to the conflict of access with other smartphone applications. As also presented in [80], Bristons used their mobile phones 17% of such 90 min per day for making phone calls. Given the microphone is an exclusive sensor that typically cannot be accessed by multiple applications at the same time, except being supported by some middleware platform for cross-sensor applications, we conservatively set the probability of occupation $P_{o}=0.17$.

**Figure 5:**
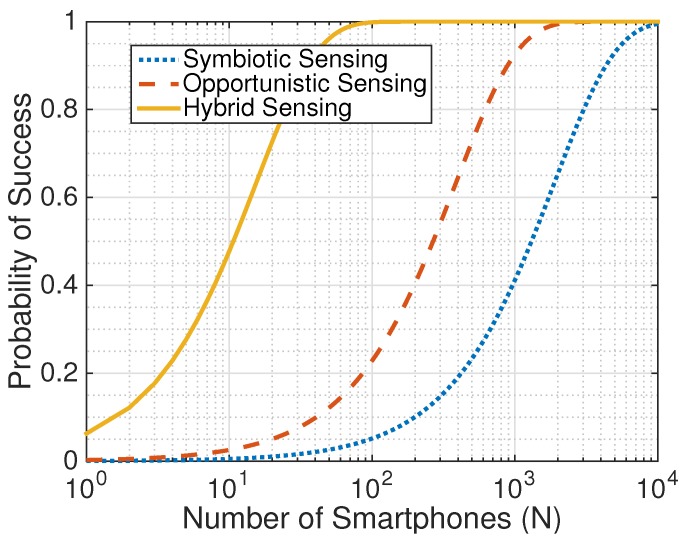
Evaluation of the NeriCell [13] honk detection application in terms of the success probability when applying opportunistic sensing (original work), symbiotic sensing, and hybrid sensing.

Using these values of $P_{s}$, $P_{c}$, and $P_{o}$, we compute the success probability of symbiotic, opportunistic, and hybrid sensing paradigms for this application, following Equations (2), (3), and (5), respectively. Since NeriCell does not specify the minimum number of smartphones to detect a bump, we set M=1. Varying the total number of smartphones *N* in the system, we plot these probabilities in Figure 5.

Figure 5 shows that for the same number of smartphones *N*, symbiotic sensing achieves a lower probability of success compared to opportunistic and hybrid sensing. In other words, if NeriCell had implemented a symbiotic sensing approach, it would have required a larger total number of smartphones to ensure that audio data is recorded when a bump is detected by accelerometers. To achieve a success probability of 1, the original implementation of NeriCell with opportunistic sensing requires at least 1774 smartphones, while symbiotic sensing requires at least 8667 smartphones, approximately five times more. This is the trade-off to the energy saving gained by using the symbiotic sensing approach. Note that in large-scale mobile sensing systems, there are thousands of smartphones participating. In such cases, the probability of success will be close to 1 even for symbiotic sensing.

In terms of energy consumption, since the HP iPAQ used in NeriCell [13] is outdated and its energy consumption is different to that of today’s smartphones, we use recent specification to derive parameters for our energy consumption models. For this, we use the energy consumption estimated by Ciman and Gaggi [81] on Samsung Galaxy i9250 smartphones using the Monsoon Power Monitor [82]. Specifically, the extra energy consumed when activating microphones is 0.4154 mAh, and that of GPS is 1.5959 mAh. Therefore, we set the Sensor Energy Consumption $e_{s}=0.4154$ mAh, and the Localization Energy Consumption $e_{l}=1.5959$ mAh. Furthermore, the power consumption in idle mode is $e_{i}=1.6582$ mAh.

For the transmission of data from the smartphones to a base station, we assume that a WiFi connection is used for its low power consumption—as low as five times less than that of other wireless interfaces such as GSM 3G or LTE [83]. As being measured by [83], the extra power consumption of WiFi when actively transferring data is 650 mAh. Thus, we set the Communication Energy Consumption $e_{c}=650$ mAh.

**Figure 6:**
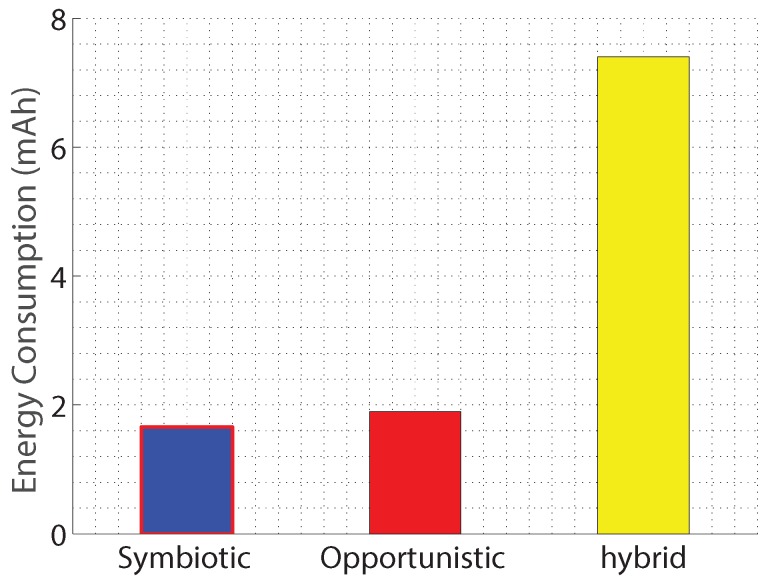
Evaluation of the NeriCell [13] honk detection application in terms of the estimated energy consumption when applying opportunistic sensing (original work), symbiotic sensing, and hybrid sensing.

Replacing these energy consumption parameters into Equations (7), (8), and (10), we obtain the expected energy consumption per device of the bump detection application when it is implemented with symbiotic, opportunistic, and hybrid sensing, respectively. The results are plotted in Figure 6. As proven in Equation (7), each smartphone installed with a symbiotic sensing paradigm consumes a constant amount of energy, which is equal to the idle power consumption such as 1.6582 mAh in our study case. Conversely, the other sensing paradigms consume more energy. For example, hybrid sensing consumes more than 7 mAh per device.

Comparing to opportunistic sensing implemented in NeriCell [13], the symbiotic sensing paradigm saves just a small amount of energy. The reason is that NeriCell continuously detects bumps with accelerometers. Then they use the detected bumps to trigger the microphones in order to conserve the battery life of the smartphones. The empirical evidence from their experiments shows that the microphones were activated by triggers only 5% of total time, which is already energy efficient. To validate this conjecture, we simulate energy consumption without the sensing condition constrained by triggers. Under such circumstances, the energy consumption per device when using the opportunistic sensing approach rises up to more than 6 mAh, which is approximately four times larger than when using the symbiotic sensing approach. Therefore, symbiotic sensing is more energy efficient than opportunistic and hybrid sensing.

### 5.2. Case Study 2: City Noise Map

In this case study, we evaluate the sensing paradigms when deployed for a noise map application using smartphones’ microphones like the Ear-Phone [47]. As the Ear-Phone application was implemented using a participatory sensing scheme, we compare this sensing paradigm with symbiotic and hybrid sensing using the models proposed in Section 4 and realistic parameters obtained from the literature.

We approximate the probability of permission $P_{p}$ as the probability of participating in cellphone surveys. For example, Brick et al. [84] contacted 4448 individuals to ask them to complete an interview via cellphones, and 1561 of them agreed to participate. Therefore, we set the probability of permission to be $P_{p}=0.3$. Additionally, in the same study, only 318 participants completed all the questions. Hence, we set the probability of user $P_{u}=318/1561\approx 0.2$.

Unlike the NeriCell system, Ear-Phone does not generate triggers to activate the microphones. Therefore, we assume that the context suitable for recording environmental sound is when the smartphone is out of pocket. Hence, we assume the probability of context $P_{c}=90/\left(24\times 60\right)=0.0625$, according to [80]. The probability that microphones are occupied is assumed to be equal the probability of usage time for making phone calls. Thus we have a probability of occupation of $P_{o}=0.17$, according to [80]. Since each smartphone has at least one microphone, we set the probability of sensor $P_{s}=1$.

**Figure 7:**
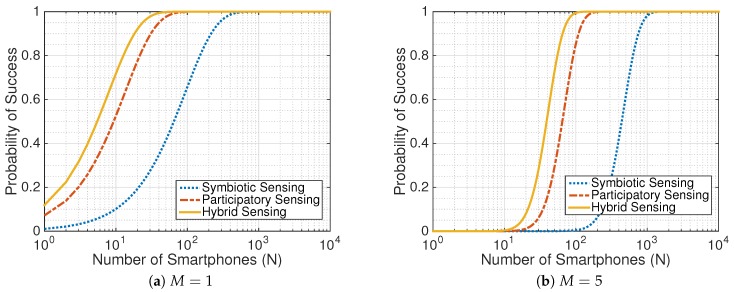
Evaluation of the Ear-Phone [47] city noise map application in terms of the success probability when applying participatory sensing (original work), symbiotic sensing, and hybrid sensing with different minimum required observations: (**a**) M=1; (**b**) M=5.

Replacing these parameter values into Equations (2), (4) and (5), we obtain the probabilities of success as plotted in Figure 7a. This figure shows that the participatory sensing paradigm achieves a higher probability of success than the symbiotic sensing paradigm for the same number of smartphones *N*. This is consistent with our theorems since participatory sensing benefits from the collaboration of users. Nevertheless, when increasing the number of participated smartphones, the success probability of symbiotic sensing also increases to 1, as high as the other sensing paradigms. Figure 7a also shows that hybrid sensing is the best choice for small-scale systems that prioritize the probability of success over energy consumption.

Recall that environmental audio samples collected from mobile phones are incomplete and inaccurate because of diversity. Ear-Phone [47] uses compressive sensing to interpolate missed samples. Another way to improve the quality of samples is to require more than one smartphone to sample the same event, i.e., M>1. Figure 7b shows the probability of success when M=5. As needing more observations, all sensing paradigms require more smartphone to deploy the sensing application. For example, to achieve the success probability of 1, the number of smartphones required by symbiotic sensing increases from 432 to 1089.

**Figure 8:**
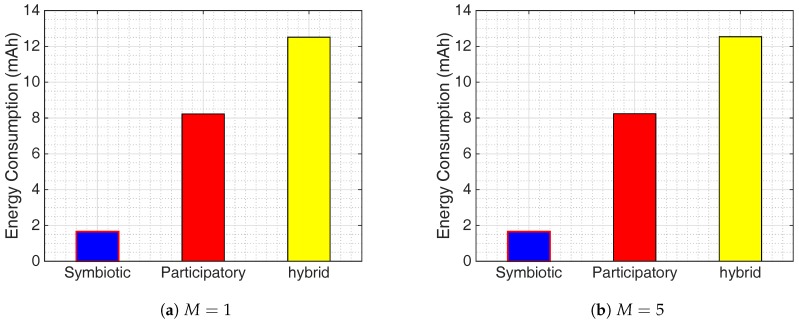
Evaluation of the Ear-Phone [47] city noise map application in terms of the expectation of estimated energy consumption when applying participatory sensing (original work), symbiotic sensing, and hybrid sensing with different minimum required observations: (**a**) M=1; (**b**) M=5.

To evaluate the energy consumption of the city noise map application, we use the same parameter values presented in the previous section. A comparison of estimated energy consumption per smartphone of symbiotic, participatory and hybrid sensing is plotted in Figure 8. This comparison shows that the average energy consumed by symbiotic sensing is only 80% of that consumed by participatory sensing.

Comparing the bar graphs in Figure 8a,b we observe that the estimated energy consumption per device does not change much for all sensing paradigms when we change *M* from 1 to 5. The reason is that the energy consumption per device heavily depends on the probability of performing sampling tasks, when the device has to activate power-hungry components and services. Nonetheless, only symbiotic sensing can yield an absolutely constant power consumption per device when varying the required observation number *M* with average energy consumption of 1.6582 mAh for both cases of *M*. For other sensing paradigms, the estimated power consumption per device indeed increases when *M* is increased. For example, the estimated power consumption per device of participatory sensing increases from 8.2287 mAh (M=1) to 8.2436 mAh (M=5), and that of hybrid sensing increases from 12.5125 mAh (M=1) to 12.5372 mAh (M=5). For the whole system, the total energy consumption for different sensing paradigms may become more distinguishable, when multiplying these incremental values with a large number of smartphones.

## 6. Discussion

In this section we will discuss remarkable points related to important features, security and privacy, and implementation feasibility of symbiotic sensing.

### 6.1. Key Features

Regardless of the absolute parameters and results of the quantitative validation in Section 5, we observe several points when considering the risk and reward in terms of the success probability and energy saving as follows.

- Sharing required resources among sensing applications, the symbiotic sensing paradigm consumes very little extra energy. In particular, it saves an amount of energy that is proportional to the probability of occupation.
- In a small-scale sensing system, the hybrid sensing paradigm would be the best choice to ensure a high success probability. However, if the application requires only a few smartphones to sense the same event simultaneously and the success probability is less prioritized, the symbiotic sensing paradigm is apromising alternative to save energy, especially when the probability of occupation is relatively high.
- In a large-scale sensing system consisting of multiple sensing applications, the symbiotic sensing paradigm is advantageous compared to other paradigms since it performs similarly or even better in terms of probability of success, whilst consuming very little extra energy for its own sensing task by sharing sensing resources.
- The advantage of the symbiotic sensing paradigm over the opportunistic sensing paradigm can be optimized by controlling the number of smartphones that need to install the sensing application. Depending on how many smartphones need to sense the same event simultaneously, the application should be deployed on a limited number of smartphones to mitigate the trade-off between energy consumption and probability of success.

### 6.2. Privacy

In general, mobile sensing involves collecting, storing, processing and fusing a huge volume of data related to daily human activities because data originates from sensors of the smartphones carried by people. In fact, smartphones can readily function as sophisticated sensor platforms, albeit not built specifically for sensing as dedicated sensing devices. Data collected by smartphones can be used to reveal contextual information of users (e.g., activities, locations, social interaction, health conditions, and behaviors). Therefore, sensed data itself needs to be encrypted and protected from untrusted viewers. The identity of the user, especially participants in the participatory sensing systems, needs to be highly secured or anonymous. Can a participant trust the sensing systems not to track their location, activities when sensing tasks execute or when they submit the reports?

Besides the aforementioned privacy issues that can happen to all kinds of sensing paradigm, sharing sensed data and derived information in symbiotic sensing has its own privacy challenges. Perhaps the most obvious concern is the security of shared data and information, especially contextual information inferred by host applications. The fact is that the information collected by a host application may be leaked through sharing with the symbiotic sensing application, although the host itself has security implemented. Another concern is the integrity of sensing applications. A sensing application that shares data needs to trust each other. If not, the symbiotic sensing paradigm cannot be implemented. It is necessary to obtain an agreement between the host and the sensing application (e.g., kinds of data can be shared, codes to decrypt data, or security for communication channels).

### 6.3. Implementation

To the best of our knowledge, the feasibility of sharing sensing resource among applications varies from operating system to operating system, and from smartphone to smartphone. For instance, Android operating systems do not allow two or more applications to simultaneously access the microphone stream since the audio recording method (MediaRecorder) is synchronized. Therefore, we implemented a symbiotic sensing service for the Android operating systems, which is a preliminary example to facilitate the implementation of symbiotic sensing in real systems. We select Android because of its proliferation over other operating systems. The symbiotic sensing service is named SENSILO (sensing silo). We publicly share this sensing service to the research community to use in large scale sensing systems. The source codes can be downloaded at [85].

**Figure 9:**
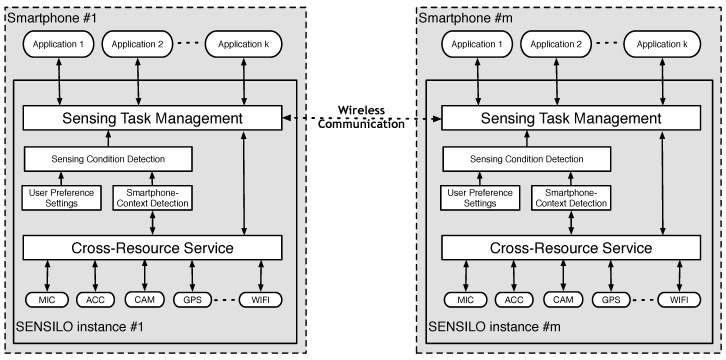
SENSILO software architecture.

Figure 9 shows the software architecture of the SENSILO service. Assume that there are *k* different sensing applications installed on *m* smartphones. These applications are assumed to have a number of overlapping sensing tasks, which should be done collaboratively through the Sensing Task Management component. In order to function without users’ awareness, these sensing tasks should be performed only if the sensing condition matches with the application design. The sensing condition is comprised of the user preferences, sampling condition, and smartphone-context condition. The user preferences are set by the user to control the user experience and privacy, for instance, which kind of sensors can be accessed and shared by the sensing application or when heavy computing tasks can be executed. The sampling condition determines if the requiring sensor is currently occupied by native applications and can be shared with sensing applications through the Cross-Resource Service. Given contextual data provided by the Cross-Resource Service, smartphone context can also detect whether it is suitable for performing sensing tasks. Note that the sensing tasks can be shared among applications installed on different smartphones via a wireless communication such as WiFi, Bluetooth, and mobile Internet.

**Figure 10:**
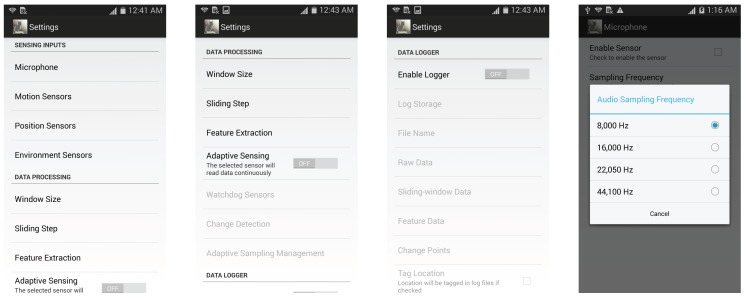
Screenshots of SENSILO settings and preference.

Figure 10 demonstrates some screenshots of the SENSILO service. In particular, SENSILO is of bound service to save sensing energy, which typically lives only while it serves a sensing application and does not run in the background continuously. In other words, when there is no sensing application opening, SENSILO kills itself. Once there is a sensing application request, SENSILO will turn on again to provide the cross-sampling service. In addition, SENSILO is capable of handling multiple requests simultaneously. To do so, we implement Android Interface Definition Language (AIDL) to perform interprocess communication (IPC), the interface for the service to communicate with its clients (sensing applications).

User preferences can be set through a graphical user interface as shown in Figure 10. The user can set the desired sampling rates, sensor types, sensor loggers, feature types, etc. For energy saving, we also implement the adaptive sampling method based on context change detection, which is presented in [86]. More details of SENSILO can be found at [85].

## 7. Conclusions

We have presented symbiotic sensing, a biology-inspired sensing paradigm for implementing energy-saving urban sensing systems based on smartphones. Since the number of smartphone applications has increased significantly while the sensing resources on smartphones are limited, the symbiotic sensing approach addresses the problem of sharing the resources as well as outcomes among the applications, similarly to the mutual relationships among living creatures in the natural world. We also proposed evaluation models to quantitatively compare the new sensing paradigm to existing ones. Through the quantitative evaluation of the models using statistical parameters and datasets from real-world sensing systems, we showed that symbiotic sensing can mitigate the energy consumption problem existing in other sensing paradigms. We also analyzed the pros and cons of smartphone-based systems using different paradigms. Although the application diversity should be considered, the symbiotic sensing paradigm is energy-efficient and scalable, showing the potential to be a better choice than others when designing large-scale systems, with an enormous number of available smartphones and applications. Moreover, the evaluation models presented in this work are useful to evaluate the potential of mobile sensing systems before deploying on a large scale. Implementing the evaluation models with specific applications will provide more conclusive evidences into that symbiotic sensing is a suitable energy-efficient approach for large-scale mobile sensing systems.

## Figures and Tables

**Table 1:** Smartphone-based Sensing Applications.

Reference	Description	Application	Paradigm	Approach ${}^{a}$	Network	Energy	Privacy
DietSense [1]	Image Scape	Personal	Participatory	C	Infrastructure	No	Yes
PerFallD [2]	Fall Detection	Personal	Opportunistic	L	Infrastructure	Yes	Yes
HealthGear [3]	Sleep Monitoring	Personal	Opportunistic	L	Infrastructure	No	No
SociableSense [4]	Speaker Recognition	Personal	Opportunistic	L	Infrastructure	Yes	No
DarwinPhones [30]	Speaker Recognition	Personal	Opportunistic	L	Ad-hoc	Yes	Yes
EyePhone [31]	Eye-based Control	Personal	Participatory	L	Infrastructure	Yes	No
CONSORTSS [32]	Health Monitoring	Personal	Participatory	L	Infrastructure	No	No
SPA [33]	Health Monitoring	Personal	Participatory	C	Infrastructure	No	Yes
BALANCE [34]	Food Advice	Personal	Participatory	C	Infrastructure	No	No
UbiFit Garden [35]	Activity Advice	Personal	Participatory	L	Infrastructure	No	Yes
HyperFit [36]	Nutrion & Exercise	Personal	Participatory	C	Infrastructure	No	No
HealthAware [37]	Obesity Tackling	Personal	Participatory	L	Infrastructure	No	No
PACER [38]	Paper Reading	Personal	Participatory	C	Infrastructure	No	No
HeartToGo [39]	Cardiovascular	Personal	Opportunistic	C	Infrastructure	No	No
EmotionSense [40]	Speaking Recognition	Social Behavior	Opportunistic	L	Infrastructure	Yes	Yes
CenceMe [5]	Sport Analysis	Social Behavior	Participatory	C	Infrastructure	No	Yes
MoVi [6]	Video Highlights	Social Behavior	Participatory	C	Infrastructure	Yes	Yes
Party [7]	Party Detection	Social Behavior	Participatory	C	Infrastructure	Yes	Yes
Crowd Counting [8]	Crowd Density	Social Behavior	Opportunistic	L	Ad-hoc	Yes	Yes
Human Mobility [41]	Mobility Pattern	Social Behavior	Opportunistic	C	Infrastructure	No	Yes
Pedestrian Flocks [42]	Flock Detection	Social Behavior	Opportunistic	C	Infrastructure	No	No
WhozThat [43]	Indoor Localization	Social Behavior	Opportunistic	C	Infrastructure	No	Yes
Blueetooth Sensing [19]	Mobility and Interactions	Social Behavior	Participatory	C	Infrastructure	Yes	No
FlierMeet [44]	Public Information Sharing	Social Behavior	Participatory	C	Infrastructure	No	No
Laermometer [45]	City Noise Map	Environmental	Participatory	C	Infrastructure	No	No
PEIR [46]	Environment Impact	Environmental	Participatory	C	Infrastructure	No	Yes
EarPhone [47]	City Noise Map	Environmental	Participatory	C	Infrastructure	Yes	Yes
MicroBlog [48]	Micro Map	Environmental	Participatory	C	Infrastructure	Yes	No
SoundSense [49]	Music Detector	Environmental	Participatory	L	Infrastructure	Yes	Yes
Citizen Journalist [7]	Citizen Journalist	Environmental	Participatory	C	Infrastructure	Yes	Yes
MobGeoSen [50]	City Noise Map	Environmental	Participatory	L	Infrastructure	No	Yes
SmartDC [9]	Human Mobility	Environmental	Opportunistic	L	Infrastructure	Yes	No
DeepEar [10]	Environmental Sound	Environmental	Opportunistic	L	Infrastructure	Yes	No
CommonSense [11]	Air Monitoring	Environmental	Participatory	C	Infrastructure	No	No
MAQS [12]	Indoor Air Monitoring	Environmental	Opportunistic	C	Infrastructure	Yes	No
iSee [51]	Event Localization	Environmental	Participatory	C	Infrastructure	No	No
Crowdsourcing [24]	Incentive Design	Environmental	Participatory	C	Infrastructure	No	No
Visibility [52]	Air Visibility	Environmental	Participatory	C	Infrastructure	Yes	Yes
NoiseTube [53]	City Noise Map	Environmental	Hybrid	C	Infrastructure	No	Yes
PEIR [46]	Environment Impact	Environmental	Hybrid	C	Infrastructure	No	Yes
BikeNET [54]	Environment Impact	Environmental	Hybrid	C	Infrastructure	No	Yes
T-Shape [55]	Environment Impact	Environmental	Hybrid	C	Infrastructure	No	Yes
Bubble Sensing [56]	Environment Impact	Environmental	Hybrid	C	Infrastructure	No	Yes
UnLoc [57]	Indoor Localization	Environmental	Opportunistic	L	Infrastructure	No	No
2Loud? [58]	City Noise Map	Environmental	Participatory	C	Infrastructure	No	Yes
Noise Monitoring [59]	City Noise Map	Environmental	Participatory	C	Infrastructure	Yes	Yes
Smart Cities [60]	City Noise Map	Environmental	Hybrid	C	Infrastructure	Yes	No
CarTel [61]	Driving Pattern	Transportation	Opportunistic	C	Delay-Tolerant	No	No
Refuelling Behavior [62]	Gas Station Placement	Transportation	Opportunistic	C	Infrastructure	No	No
GreenGPS [63]	Fuel Efficient Routes	Transportation	Participatory	C	Infrastructure	No	No
ParkNet [64]	Road-side Parking	Transportation	Opportunistic	C	Infrastructure	No	No
Travel Time [65]	Congestions Detection	Transportation	Opportunistic	C	Infrastructure	No	Yes
Bus Waiting [66]	Bus Arrival Prediction	Transportation	Participatory	C	Infrastructure	Yes	No
Railway Trip [67]	Passenger Congestion	Transportation	Opportunistic	C	Infrastructure	No	No
Crowd Density [68]	Crowd Density	Transportation	Opportunistic	C	Infrastructure	No	No
Pedestrian Flows [69]	Crowd Congestion	Transportation	Participatory	C	Infrastructure	Yes	No
VTrack [70]	Route Planning	Transportation	Participatory	C	Infrastructure	Yes	No
NeriCell [13]	Bump Detection	Transportation	Opportunistic	L	Infrastructure	Yes	Yes
Road Bump [7]	Bump Detection	Transportation	Opportunistic	C	Infrastructure	Yes	Yes
AnomySense [71]	Lost&Found	Transportation	Opportunistic	C	Infrastructure	Yes	Yes
SmartRoad [14]	Traffic Regulator Detection	Transportation	Participatory	C	Infrastructure	Yes	No
PublicSense [15]	Public Facility Management	Transportation	Participatory	C	Infrastructure	No	No
CrowdWatch [16]	Sidewalk Obstacle Detection	Transportation	Opportunistic	L	Infrastructure	No	No
Road Crack [72]	Road Crack Monitoring	Transportation	Participatory	C	Infrastructure	No	No

^a^ C: centralized, collected data are mainly processed at a central sever; L: local, collected data are mainly processed on mobile devices.
